# The unmet promise: a critical review of antioxidant strategies in myocardial ischemia-reperfusion injury and the path towards precision medicine

**DOI:** 10.3389/fphar.2025.1693441

**Published:** 2025-11-10

**Authors:** Hao Chen, Yutao Tang, Pan Ren, Wenbin Wu

**Affiliations:** 1 Hospital of Chengdu University of Traditional Chinese Medicine, Chengdu, China; 2 The Affiliated Traditional Chinese Medicine Hospital, Southwest Medical University, Luzhou, China

**Keywords:** myocardial ischemia, oxidative stress, ischemia-reperfusion injury, endogenous antioxidant defense, translational challenges, redox signaling, therapeutic strategies, precision medicine

## Abstract

Myocardial ischemia-reperfusion injury (MIRI) remains a major clinical challenge, with oxidative stress as a key driver. Despite extensive preclinical promise, antioxidant therapies have consistently failed in clinical translation. This critical review deconstructs this translational gap, which stems from the nuanced complexity of redox biology, inappropriate therapeutic timing, and patient heterogeneity. We argue that overcoming these hurdles requires a paradigm shift from broad antioxidant supplementation to precision medicine. This approach involves enhancing endogenous defense mechanisms, leveraging targeted drug delivery, and developing multi-modal strategies. Ultimately, integrating dynamic biomarkers, multi-omics, and artificial intelligence to tailor treatments to individual patient profiles holds the key to finally fulfilling the promise of effectively managing MIRI.

## 1 Introduction

Myocardial ischemia (MI) is a critical global health concern with high prevalence and mortality (Sayed et al., 2025). The global burden of ischemic heart disease is projected to rise dramatically, remaining the leading cause of cardiovascular death and accounting for a projected 20 million deaths by 2050 (Chong et al., 2025). MI is defined as the inadequate blood supply to the myocardium, which, if severe and prolonged, can result in myocardial infarction (Aslam et al., 2025). Thus, sophisticated and targeted therapeutic strategies are imperative to mitigate this disease burden.

Oxidative stress represents a fundamental pathophysiological mechanism implicated in myocardial ischemia-reperfusion injury (MIRI) (Luo et al., 2023). This pivotal process is marked by a dynamic and often overwhelming increase in reactive oxygen species (ROS). While ROS generation is modest during the ischemic phase due to oxygen deprivation (Sen et al., 2024), a substantial and rapid “burst” occurs upon the sudden reintroduction of oxygen during reperfusion (Xiang et al., 2021; Dhalla et al., 2022; Guo et al., 2024). Principal contributors to this oxidative burst include dysfunctional mitochondria during reoxygenation, activated NADPH oxidases, and the infiltration of inflammatory cells (Liu N. et al., 2021). The excessive generation of ROS catalyzes a cascade of deleterious events within cardiomyocytes and the surrounding tissue. The oxidative burst is instrumental in initiating inflammatory pathways, notably the activation of the NLRP3 inflammasome, which subsequently results in the secretion of potent pro-inflammatory cytokines such as IL-1β and IL-18 (Chen et al., 2025). These oxidative and inflammatory signals interact synergistically, contributing to irreversible myocardial damage characterized by lipid peroxidation, protein oxidation, DNA damage, and various forms of cell death. This cascade ultimately impairs cardiac function and leads to adverse clinical outcomes (Yu et al., 2021; Duan D. et al., 2023). Consequently, a comprehensive understanding of oxidative stress and its downstream effects is essential for the development of effective therapeutic strategies aimed at mitigating MIRI and improving patient prognosis.

In recent decades, a myriad of antioxidant strategies, ranging from direct exogenous supplementation to modulation of endogenous defense mechanisms, have been extensively investigated to alleviate MIRI. While preclinical studies have consistently demonstrated promising cardioprotective effects, the clinical application of numerous antioxidant therapies has, paradoxically, produced largely inconsistent and often disappointing outcomes, leading to an ‘unmet promise’ in this critical field (Lonn et al., 2005; Rodrigo et al., 2014). This discrepancy highlights substantial challenges, including poor drug delivery, suboptimal timing, patient heterogeneity, and the nuanced dual roles of ROS.

This review seeks to conduct a critical examination of the complex role that oxidative stress plays in the pathogenesis of MI and MIRI, moving beyond a mere summary of existing knowledge. We will analytically synthesize both current and emerging therapeutic strategies, with a particular focus on deconstructing the reasons behind their limited clinical success. Crucially, we aim to delineate a forward-looking ‘path towards precision medicine’ within this vital field, particularly by leveraging insights from individual patient oxidative stress profiles, robust biomarker development, and advanced technological integration to tailor treatment strategies and ultimately improve patient prognosis.

## 2 Pathophysiological mechanisms of oxidative stress in MIRI

### 2.1 Concept and dynamic changes of oxidative stress in MIRI

**FIGURE 1:**
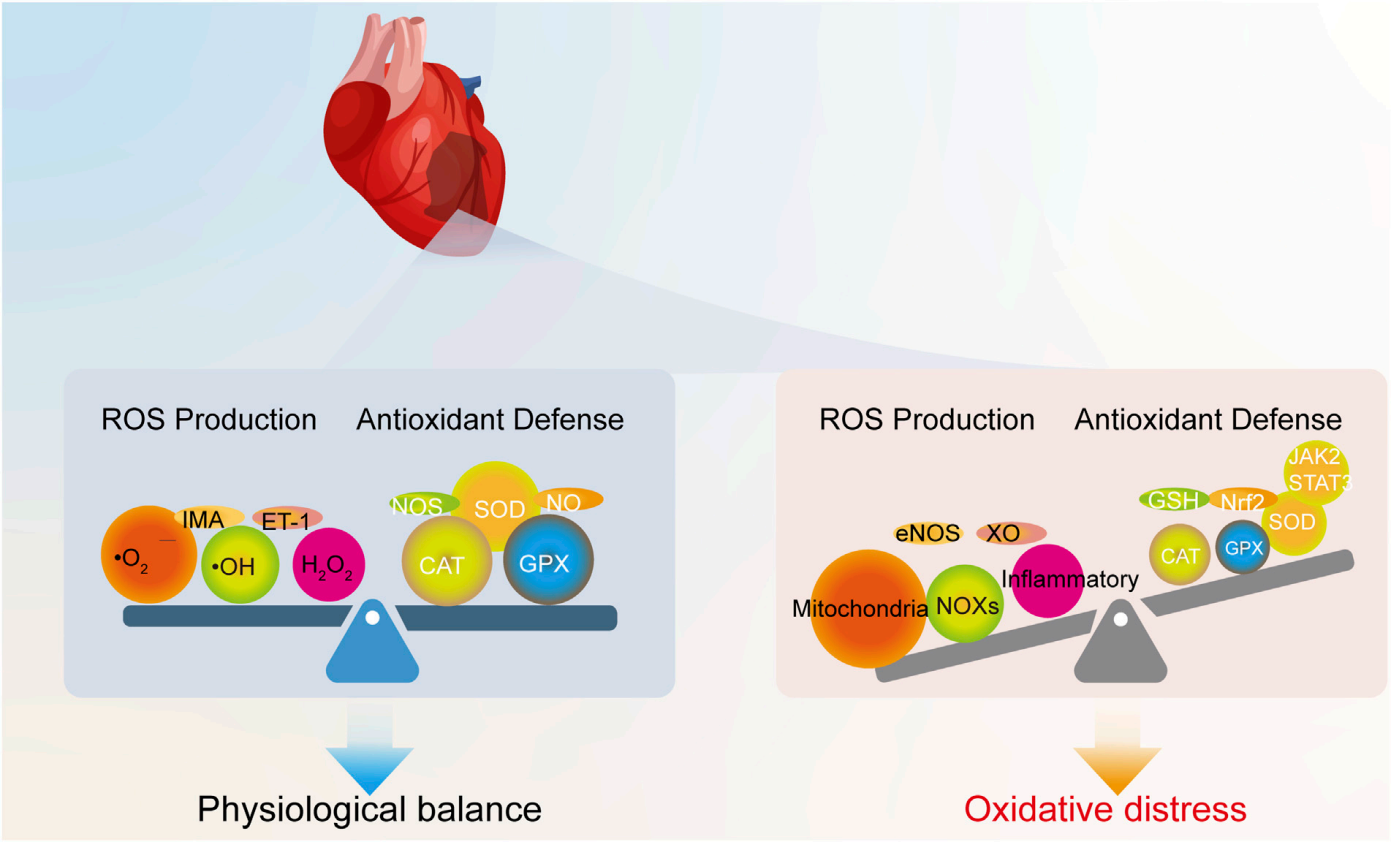
Physiological redox balance versus oxidative stress in the heart. Under physiological conditions, ROS generated from normal metabolism are neutralized by endogenous antioxidant systems. In contrast, oxidative stress occurs when excessive ROS production from various sources overwhelms these defense mechanisms.

Oxidative stress is fundamentally defined as an imbalance between the production of ROS and the capacity of the body’s antioxidant defense systems to neutralize them (Figure 1) (Jaganjac et al., 2022). ROS are highly reactive oxygen-derived molecules (e.g., superoxide anion •O_2_^−^, hydroxyl radical •OH, hydrogen peroxide H_2_O_2_). Although these species are generated during normal cellular metabolism and participate in essential physiological processes like signal transduction and gene expression at low concentrations (Krishnamurthy et al., 2024), their excessive accumulation or insufficient scavenging leads to oxidative stress, causing damage to vital biomolecules and cellular structures.

**FIGURE 2:**
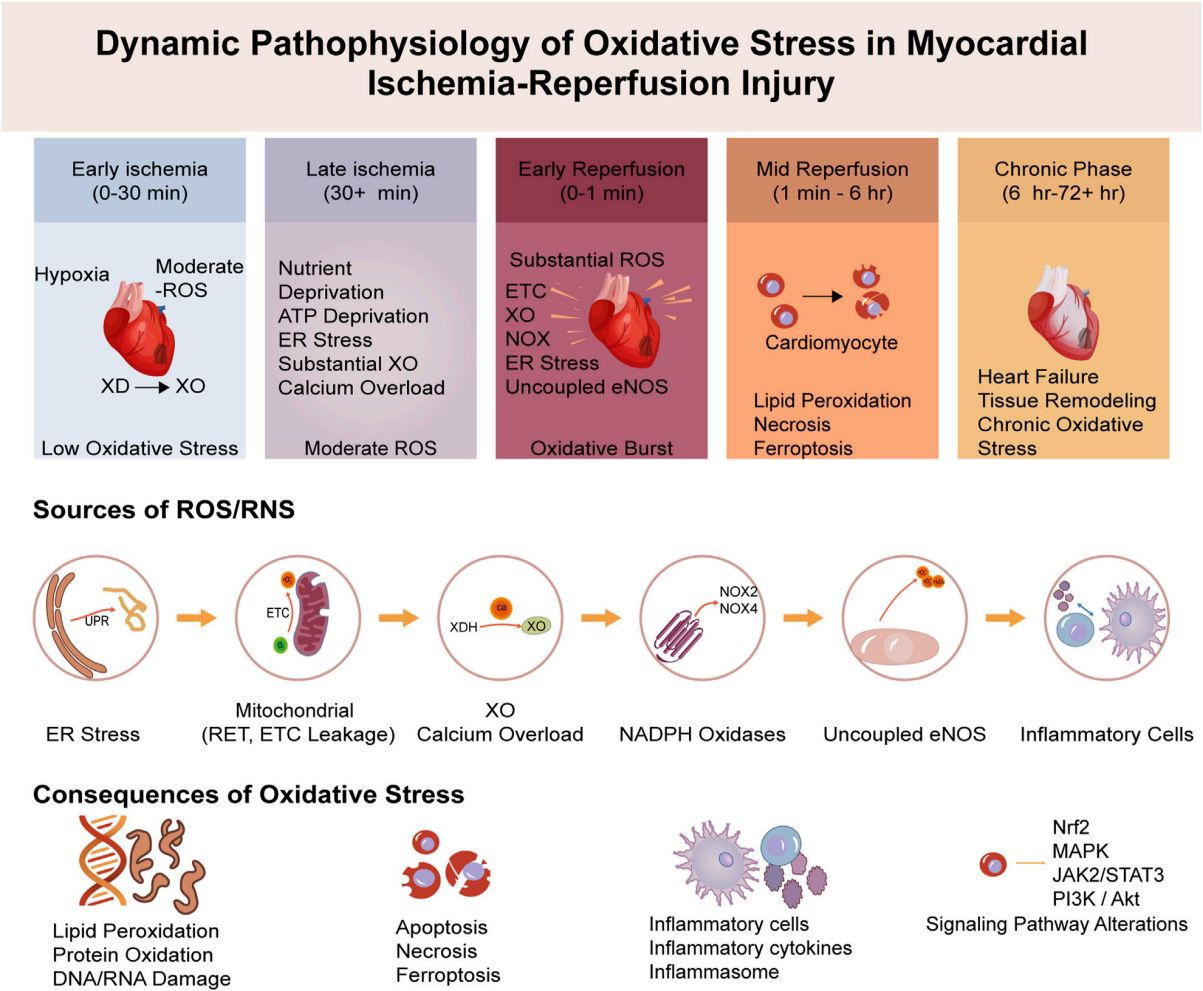
Timeline of pathological events in MIRI. The progression from ischemia (low ROS) to a massive oxidative burst upon reperfusion, which triggers downstream inflammation, cell death, and adverse remodeling.

In the specific pathological context of MIRI, oxidative stress becomes particularly aggressive and dynamic. The generation of ROS in the myocardium is dynamic, undergoing significant changes throughout the phases of ischemia and reperfusion (Alizadehasl et al., 2019), as summarized in Figure 2. During the ischemic phase, severe hypoxia and nutrient deprivation impair mitochondrial function, leading to a modest increase in ROS production due to limited electron leakage from the dysfunctional mitochondrial electron transport chain (ETC.) (Sen et al., 2024). However, the lack of oxygen and substrates generally keeps ROS generation relatively lower compared to the subsequent phase.

**FIGURE 3:**
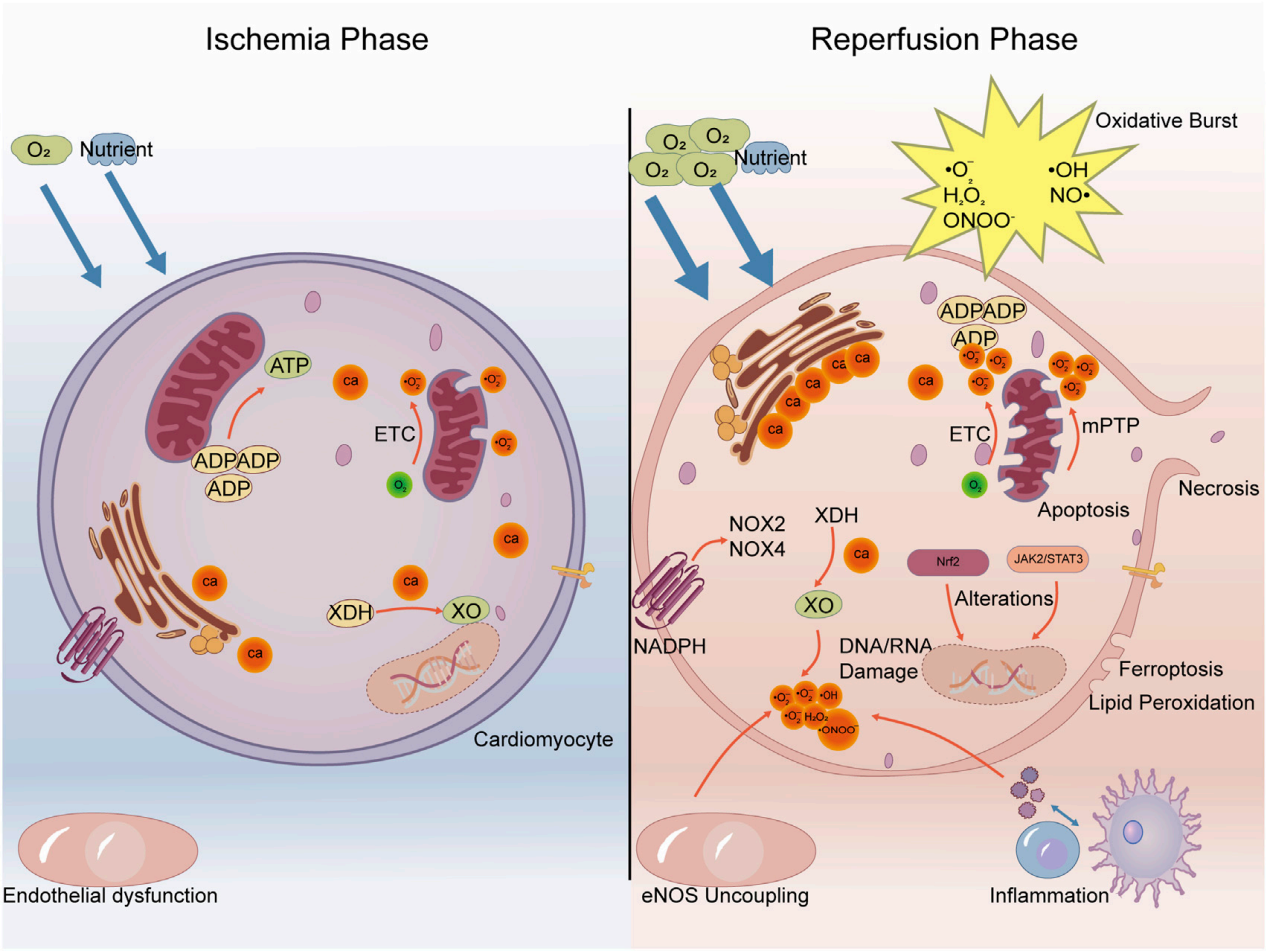
The pathophysiological cascade of MIRI. Ischemic triggers lead to a reperfusion-induced oxidative burst. This overwhelms endogenous defenses, causing direct molecular damage and activating downstream inflammation, cell death, and signaling alterations, which collectively drive myocardial injury.

The most damaging oxidative insult occurs upon reperfusion, where the sudden reintroduction of oxygen paradoxically triggers a massive burst of ROS and reactive nitrogen species (RNS). This concept, widely known as the “oxidative burst,” was foundationally established in seminal studies demonstrating that post-ischemic tissue injury is driven by oxygen-derived free radicals generated upon reoxygenation (Granger et al., 1981; Epstein and McCord, 1985) and has since been extensively characterized (Guo et al., 2024). This burst is a critical mediator of reperfusion injury, overwhelming the compromised antioxidant defense systems and causing widespread cellular damage. This dramatic increase is exacerbated by factors unique to the reperfused myocardium, including calcium overload, rapid restoration of mitochondrial electron transport, activation of enzymes like NADPH oxidase and xanthine oxidase, and the infiltration of inflammatory cells that release a wide array of oxidants (Liu N. et al., 2021; Quarato et al., 2022) (Figure 3).

#### 2.1.1 Mitochondrial dysfunction and reoxygenation

Mitochondria are the primary site of oxygen consumption and a major source of ROS in the heart. During ischemia, mitochondrial, ETC., components become damaged and partially reduced (Sen et al., 2024). Upon reperfusion, the sudden availability of oxygen allows rapid, but often inefficient, electron flow through the damaged, ETC. This leads to significant electron leakage, particularly from Complexes I and III, which directly reduces oxygen to superoxide anion (•O_2_^−^) (Xue et al., 2020; Liu N. et al., 2021).

A key mechanism of mitochondrial ROS production upon reperfusion is reverse electron transport (RET) at Complex I. During ischemia, succinate accumulates significantly within the mitochondrial matrix. Upon reperfusion, this accumulated succinate is rapidly oxidized by succinate dehydrogenase (SDH, Complex II), leading to a highly reduced coenzyme Q (CoQ) pool. Subsequently, driven by a high mitochondrial membrane potential (ΔΨm) and the reduced CoQ pool, electrons are “pushed back” through Complex I, where they react with molecular oxygen, leading to a burst of superoxide anion production (•O_2_^−^) (Chouchani et al., 2014). The re-energization of the mitochondria with substrates like succinate can lead to a high proton-motive force, driving electrons backward through Complex I, which then reduces oxygen to superoxide at a high rate (Casey et al., 2025). Furthermore, ischemia-induced mitochondrial calcium overload and permeability transition pore (mPTP) opening exacerbate mitochondrial dysfunction and ROS production (Griffiths and Halestrap, 1993; Quarato et al., 2022).

#### 2.1.2 Calcium overload

Elevated intracellular and mitochondrial calcium levels, a hallmark of MIRI, contribute to ROS generation through multiple mechanisms. Calcium activates proteases that convert XDH to XO, disrupts mitochondrial function leading to increased electron leakage, and can activate other ROS-producing enzymes (Huang et al., 2020).

#### 2.1.3 Xanthine oxidase

During ischemia, ATP is degraded to hypoxanthine and xanthine due to energy depletion. The enzyme xanthine dehydrogenase (XDH), normally producing NADH, is converted to its oxidase form (XO) through calcium-activated proteases or oxidative modification (Kusano et al., 2023). Upon reperfusion, with the reintroduction of oxygen, XO utilizes the accumulated hypoxanthine/xanthine and oxygen to produce large amounts of uric acid and •O_2_^−^ (and subsequently H_2_O_2_) (Liu N. et al., 2021). This pathway is a significant contributor to the early reperfusion oxidative burst.

#### 2.1.4 NADPH oxidases

NADPH Oxidases (NOXs) are multi-subunit enzymes that generate •O_2_^−^ from oxygen and NADPH (Kračun et al., 2025). Various NOX isoforms (e.g., NOX2, NOX4) are expressed in cardiomyocytes, endothelial cells, and fibroblasts. Their activity is significantly upregulated during both ischemia and, more prominently, reperfusion in response to stimuli like angiotensin II, cytokines, and mechanical stress (Quarato et al., 2022). Notably, the infiltration and activation of inflammatory cells (neutrophils, macrophages) during reperfusion introduce a substantial source of ROS *via* their highly active phagocytic NOX (NOX2) system (Li et al., 2025).

#### 2.1.5 Endothelial nitric oxide synthase (eNOS) uncoupling

Endothelial cells produce nitric oxide (NO•) *via* eNOS, which is crucial for vasodilation and cardioprotection. However, under conditions of oxidative stress and substrate/cofactor (like tetrahydrobiopterin, BH4) depletion during ischemia/reperfusion, eNOS can become “uncoupled.” In this state, it produces •O_2_^−^ instead of NO•. The reaction between simultaneously produced NO• and •O_2_^−^ rapidly forms the highly reactive and damaging reactive nitrogen species (RNS), peroxynitrite (ONOO^−^) (Liu N. et al., 2021).

#### 2.1.6 Inflammatory cell infiltration and activation

As mentioned regarding NOX, the recruitment and activation of neutrophils and macrophages to the injured myocardium during reperfusion is a major source of sustained ROS production. These cells release a barrage of oxidants as part of their immune response, contributing significantly to tissue damage and the inflammatory cascade (Zhang H. et al., 2021; Li et al., 2025).

**FIGURE 4:**
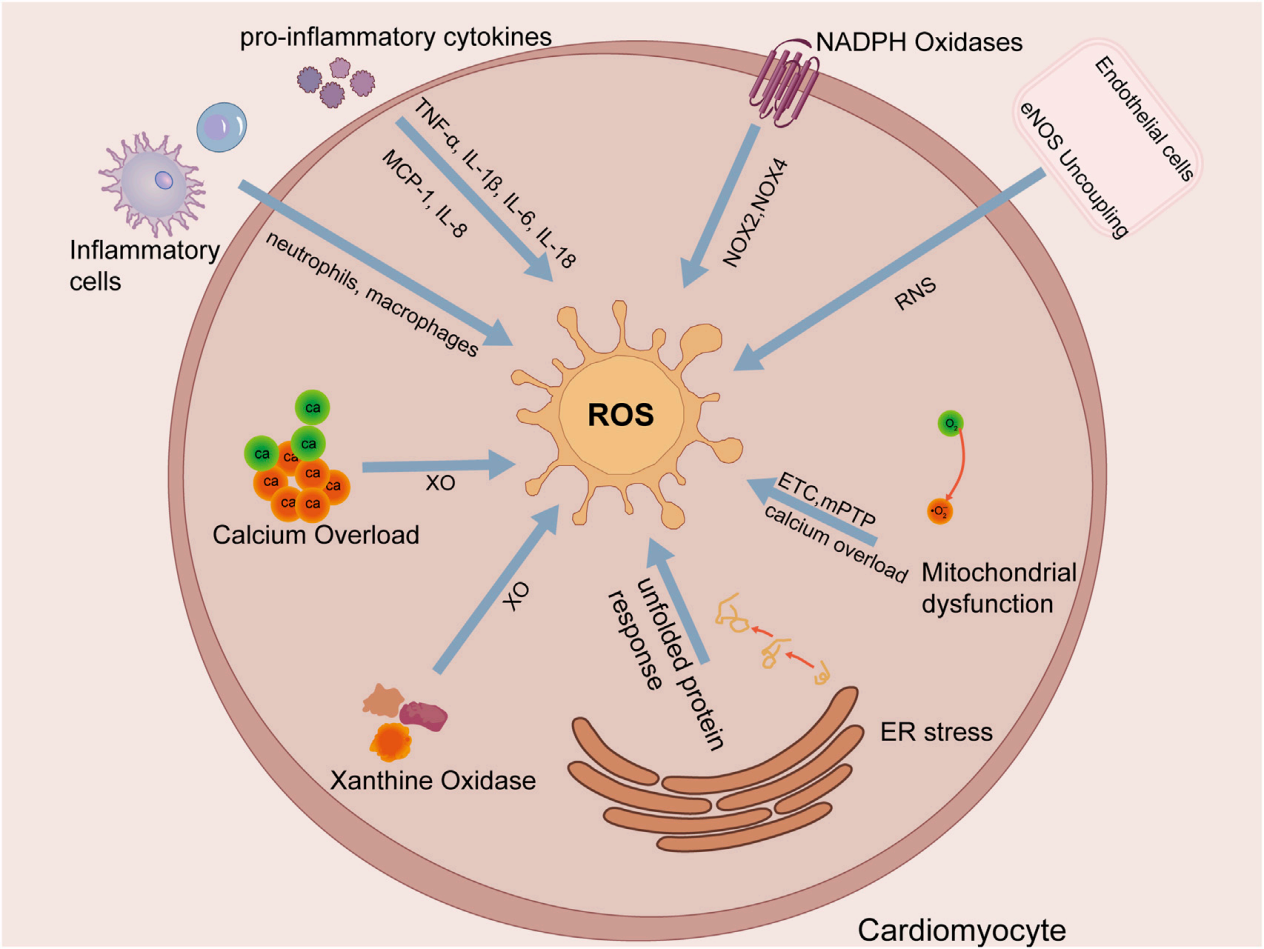
Primary sources of ROS in cardiomyocytes during MIRI. Key sources include mitochondria, NADPH oxidases, uncoupled eNOS, xanthine oxidase, ER stress, and inflammatory cells.

In addition to these major sources, the endoplasmic reticulum can contribute to the ROS burden (Gong et al., 2025). The accumulation of misfolded proteins during ischemia triggers the unfolded protein response (UPR). While initially adaptive, prolonged or severe ER stress leads to excessive ROS production, further exacerbating cellular injury (Zhang et al., 2025). The major cellular sources contributing to this ROS burst are summarized in Figure 4.

### 2.2 Dual roles of ROS: from physiological signaling to the critical pitfall of redox hormesis

The role of ROS in MIRI is not a simple dichotomy of beneficial signaling *versus* pathological damage; it is a complex, concentration-dependent phenomenon that is fundamental to understanding therapeutic failures. At low, physiological concentrations, ROS function as essential second messengers in a process known as redox signaling. Species like H_2_O_2_ and NO• participate in vital cellular cascades that regulate cell growth, differentiation, and survival by reversibly oxidizing specific protein targets, thereby maintaining cellular homeostasis (Gammella et al., 2016; Krishnamurthy et al., 2024). In stark contrast, the massive and uncontrolled “oxidative burst” during reperfusion overwhelms these delicate regulatory systems, causing widespread, indiscriminate damage to lipids, proteins, and nucleic acids, which directly drives myocardial injury (Fedoreyeva, 2024).

Further complicating this picture is the crucial concept of redox hormesis, which reveals that the biological response to ROS follows a non-linear, often U-shaped, curve. This model posits that while both very low and very high levels of ROS can be detrimental, a moderate and transient increase in ROS can be profoundly protective (Wang et al., 2014). This mild oxidative challenge acts as a trigger, activating powerful endogenous defense mechanisms, most notably the Nrf2 pathway. This process effectively pre-conditions the cell to withstand subsequent, more severe insults, a mechanism that mirrors the protective effects of ischemic preconditioning (Wu et al., 2023). Therefore, a moderate ROS signal is not an early sign of damage but a critical catalyst for cellular resilience.

This non-linear relationship explains why non-specific, high-dose antioxidant therapies have persistently failed in clinical trials. Aiming for complete ROS eradication, these strategies disrupt this vital hormetic balance and may inadvertently cause harm. By indiscriminately scavenging all ROS, these therapies likely blunt the essential, moderate signaling required to activate the heart’s own powerful intrinsic defense pathways. In doing so, they may render the myocardium more vulnerable, providing a stark example of a therapeutic strategy that, while mechanistically logical on the surface, fails to account for the underlying biological complexity. This critical therapeutic pitfall underscores why future strategies must shift from broad ROS elimination to the nuanced restoration of redox homeostasis.

### 2.3 Dysregulation and failure of endogenous antioxidant defense mechanisms

Under physiological conditions, the myocardium maintains redox homeostasis through a robust endogenous antioxidant defense system, comprising enzymatic components like SOD, Catalase (CAT), and Glutathione Peroxidases (GPxs), as well as non-enzymatic antioxidants such as glutathione (GSH) and thioredoxin (Kumar et al., 2022). These systems are crucial for neutralizing physiological levels of ROS and preventing oxidative damage. However, during MIRI, this crucial defense system is severely overwhelmed and dysregulated, rendering it incapable of counteracting the massive oxidative burst (Jiang et al., 2023).

This multifaceted dysregulation involves the rapid depletion and inactivation of key antioxidants and enzymes due to excessive ROS production (Karaduleva et al., 2016; Korshunov and Imlay, 2024). Furthermore, factors like calcium overload can interfere with the function of antioxidant enzymes, and epigenetic modifications can negatively impact the expression of antioxidant genes. Crucially, key regulatory signaling pathways that normally bolster antioxidant defenses are also disrupted or insufficiently activated to cope with the overwhelming stress. The Nuclear factor erythroid 2-related factor 2 (Nrf2) pathway, a master regulator of antioxidant gene expression (Shen et al., 2019), is activated by oxidative stress as a protective feedback mechanism (Itoh et al., 1997; 1999). Similarly, the JAK2/STAT3 pathway is known to promote cardiomyocyte survival and upregulate anti-apoptotic and antioxidant proteins in response to stress (Xiong et al., 2021). Upon activation, JAK2 phosphorylates STAT3, leading to STAT3 dimerization, translocation to the nucleus, and binding to target gene promoters. In the context of MIRI, JAK2/STAT3 activation is often considered a protective response in the acute phase, promoting cardiomyocyte survival by upregulating anti-apoptotic proteins (like Bcl-xL and Mcl-1) and antioxidant enzymes (Liu P. et al., 2021; Xiong et al., 2021). While Nrf2 and JAK2/STAT3 are indeed activated as protective responses during MIRI, the overwhelming and dynamic nature of the oxidative insult often impairs their full protective capacity or overwhelms the defense mechanisms they regulate, leading to a functional failure in restoring redox balance. This failure of the endogenous defense system to restore redox balance is a critical step that allows oxidative stress to exert its full pathological effects.

In summary, the overwhelming production of ROS during MIRI, coupled with the depletion, inactivation, and dysregulation of the intrinsic antioxidant defense mechanisms and their regulatory pathways, leads to a state of severe redox imbalance. This failure to effectively neutralize oxidants is a prerequisite for the subsequent cascade of damaging downstream events that drive myocardial injury.

### 2.4 Oxidative stress-mediated downstream pathological consequences

With the endogenous antioxidant defense system overwhelmed and dysregulated (as discussed in Section 2.3), the excessive and unchecked accumulation of ROS during MIRI triggers direct and widespread damage at the molecular level. This molecular carnage manifests as: **(i) lipid peroxidation**, which disrupts membrane integrity and is a key driver of ferroptosis (Yu et al., 2021; Fujii and Imai, 2024); **(ii) protein oxidation,** leading to conformational changes and loss of function in enzymes and structural proteins (Li Y. et al., 2022; Duong et al., 2024); and **(iii) DNA/RNA damage,** which can activate cell death pathways (Seixas et al., 2021; Cardinale et al., 2022). Collectively, this widespread molecular damage serves as the direct trigger for the major downstream pathological consequences of inflammation and cell death.

#### 2.4.1 Inflammation

Oxidative stress is a potent trigger and amplifier of the inflammatory response in the ischemic and reperfused myocardium. Unchecked ROS can directly activate key pro-inflammatory transcription factors, such as Nuclear Factor-kappa B (NF-κB) and Activator Protein 1 (Yusuf et al., 2025). Activation of NF-κB, for instance, promotes the transcription of genes encoding a wide array of pro-inflammatory mediators, including cytokines (e.g., TNF-α, IL-1β, IL-6, IL-18) and chemokines (e.g., MCP-1, IL-8) (Liu et al., 2020). These secreted factors attract and activate inflammatory cells, such as neutrophils and macrophages, to the site of injury (Liang et al., 2025). Furthermore, oxidative stress is a critical activator of inflammasomes, particularly the NLRP3 inflammasome (Jomova et al., 2023), leading to the release of potent pro-inflammatory cytokines that drive sterile inflammation and contribute significantly to tissue damage (Chen et al., 2025). The interplay between unchecked oxidative stress and inflammation forms a vicious cycle, amplifying injury (Zhang H. et al., 2021; Li et al., 2025).

#### 2.4.2 Cell death

Oxidative stress is a major inducer of cardiomyocyte death in MIRI, contributing significantly to the loss of functional myocardium. Multiple forms of cell death are involved, including apoptosis, necrosis, and ferroptosis. Excessive ROS can trigger the mitochondrial pathway of apoptosis by disrupting mitochondrial membrane potential and releasing pro-apoptotic factors (Luis-García et al., 2021; Wolf et al., 2022). Oxidative stress also contributes to necrosis by damaging cell membranes through lipid peroxidation and exacerbating calcium dysregulation, leading to cell swelling and lysis (Munoz et al., 2017; Xue et al., 2020; Fujii and Imai, 2024). Increasingly recognized, ferroptosis is a distinct form of regulated cell death driven by iron accumulation and lethal lipid peroxidation (Yu et al., 2021; Fujii and Imai, 2024). Oxidative stress plays a central role by initiating lipid peroxidation chains and inhibiting the activity of glutathione peroxidase 4 (GPX4), a key enzyme preventing ferroptosis (Xu et al., 2021).

#### 2.4.3 Signaling pathway alterations

Beyond activating inflammatory and cell death cascades, overwhelming oxidative stress pathologically alters numerous other intracellular signaling pathways crucial for myocardial function and survival. The MAPK pathways (including p38, JNK, and ERK) are highly sensitive to oxidative stress and play diverse, often detrimental, roles in MIRI, mediating inflammation, apoptosis, and maladaptive stress responses (Xu et al., 2015). The Phosphoinositide 3-Kinase (PI3K)/Akt pathway, while typically pro-survival, can be complexly modulated by pathological oxidative stress, impacting cardiomyocyte viability and metabolism in ways that may not always be protective (Lu et al., 2023). While Nrf2 and JAK2/STAT3 have protective roles, severe oxidative stress can also lead to their dysregulation or interaction with other pathways in ways that contribute to later-stage remodeling or inflammation (Mahdiani et al., 2022; Ye et al., 2024). These widespread alterations in signaling networks contribute to the overall pathological phenotype of the injured myocardium.

In summary, the failure of the endogenous antioxidant defense system allows the massive oxidative insult to trigger a complex array of downstream pathological consequences, including robust inflammatory responses, multiple forms of cardiomyocyte death, and widespread disruption of vital signaling networks. These interconnected events collectively contribute to the extent of myocardial damage and ultimately determine functional recovery.

## 3 Therapeutic strategies targeting oxidative stress

### 3.1 Exogenous antioxidant supplementation

Direct administration of exogenous antioxidants, while intuitively appealing, is a significant component of the ‘unmet promise’ in MIRI. Despite extensive preclinical support, clinical trials of these agents have yielded largely inconsistent and disappointing outcomes (summarized in Table 1), highlighting fundamental translational challenges.

**TABLE 1:** Clinical trials evaluating oxidative stress-related interventions in MI.

Drug	Phase	Clinical setting	Main results	Sample size	Intervention period	Experimental groups	References
Direct antioxidants							
Vitamin e	II	Chronic Secondary Prevention	Reduced markers of myocardial injury	20	During surgery	Vitamin e vs. Control	Canbaz et al. (2003)
Vitamin e	III	Metabolic modifiers	No significant cv event reduction; potential heart failure risk	9,541	7 years	Vitamin e vs. Placebo	Lonn et al. (2005)
Vitamin c + vitamin e	II	Acute peri-PCI	Post-pci, myocardial infarct size significantly reduced and endothelial function improved	40	Single dose pre-PCI	Vitamin c + vitamin e vs. Placebo	Rodrigo et al. (2014)
Edaravone	II	Acute peri-PCI	Reduced reperfusion injury and decreased ck-mb levels	101	72 h	Edaravone vs. Placebo	Tsujita et al. (2006)
Nac	II	Stable angina	Reduced markers of ischemia-reperfusion injury during surgery	20	During surgery	Nac vs. Control	Orhan et al. (2006)
Nac	II	Acute peri-PCI	Significant reduction in infarct size and improved myocardial salvage rate	112	2 days	Nac (nac + nitroglycerin) vs. Placebo	Pasupathy et al. (2017)
Melatonin	II	Acute peri-PCI	No significant improvement in infarct size or lvef	146	Single dose at PCI	Melatonin vs. Placebo	Dominguez-Rodriguez et al. (2017)
Coenzyme q10	III	Chronic HF	Improved survival in patients	420	2 years	Coenzyme q10 vs. Placebo	Mortensen et al. (2014)
Selenium + coenzyme q10	III	Chronic Secondary Prevention	Combined supplementation significantly improved serum sulfhydryl group levels	434	48 months	Selenium + coenzyme q10 vs. Placebo	Dunning et al. (2023)
Selenium + coenzyme q10	III	Chronic Secondary Prevention	Combined supplementation improved cardiac function and reduced cardiovascular mortality	443	48 months	Selenium + coenzyme q10 vs. Placebo	Alehagen et al. (2022)
Nrf2 activators							
Sulforaphane	II	Chronic HF	Results pending	200	24 weeks	Sulforaphane vs. Placebo	Nct05408559
Anti-necrosis agents							
Erythropoietin	II	Acute peri-PCI	No improvement in LVEF at 6 months in STEMI patients after PCI.	198	Single dose post-PCI	Epo vs. Placebo	Minamino et al. (2018)
Erythropoietin	II	Chronic Secondary Prevention	Short-term epo minimally affected monocyte transcription profiles	18	18 days	Epo vs. no epo	Jie et al. (2023)
Bendavia	II	Acute peri-PCI	No reduction in infarct size	297	Single dose pre-PCI	Bendavia vs. Control	Hortmann et al. (2019)
Alpha-lipoic acid	III	Metabolic modifiers	Alpha-lipoic acid improved oxidative stress marker	60	6 months	Ala vs. Control	Abdel Hamid et al. (2022)
Cyclosporine	II	Acute peri-PCI	40% reduction in infarct size with cyclosporine	58	Before PCI	Cyclosporine vs. Control	Theruvath and Lemasters (2008)
Cyclosporine	III	Acute peri-PCI	No improvement in 1-year clinical endpoints or heart failure incidence	970	Single dose pre-PCI	Cyclosporine vs. Placebo	Cung et al. (2015)
Febuxostat	III	Chronic Secondary Prevention	Reduced cardiovascular event risk in patients with gout	6,128	Long-term oral	Febuxostat vs. Allopurinol	Choi et al. (2021)
Anti-inflammatory agents							
Darapladib	III	Acute peri-PCI	No reduction in the risk of major coronary events	13,026	2.5 years	Darapladib vs. Placebo	O’donoghue et al. (2014)
Canakinumab	III	Chronic Secondary Prevention	Significant reduction in the risk of recurrent mi/stroke/cardiovascular death	10,061	∼3.7 years	Canakinumab vs. Placebo	Ridker et al. (2019)
Methotrexate	III	Chronic Secondary Prevention	No reduction in cardiovascular event risk	4,786	Weekly oral (2–3 years)	Methotrexate vs. Placebo	Ma et al. (2025)
Colchicine	III	Chronic Secondary Prevention	Significant reduction in ischemic cardiovascular composite endpoint	4,745	∼22.6 months (0.5 mg daily)	Colchicine vs. Placebo	Tardif et al. (2019)
Colchicine	III	Chronic Secondary Prevention	Reduced cardiovascular events	5,522	29 months (median)	Colchicine vs. Placebo	Nidorf et al. (2020)
Tocilizumab	II	Acute peri-PCI	Significantly higher myocardial salvage index but no significant difference in final infarct volume post-PCI.	199	Single dose pre-PCI	Tocilizumab vs. Placebo	Broch et al. (2021)
Anakinra	II	Acute peri-PCI	Significant reduction in 1-year death or new-onset heart failure in stemi patients	139	14 days	Anakinra vs. Placebo	Abbate et al. (2022)
SGLT2 inhibitors							
Dapagliflozin	IV	Metabolic modifiers	Dapagliflozin improved endothelial function	97	12 weeks	Metformin + dapagliflozin, metformin + glibenclamide	Sposito et al. (2021)
Dapagliflozin	IV	Metabolic modifiers	Dapagliflozin improved endothelial function and reduced oxidative stress	80	16 weeks	Metformin + dapagliflozin vs. Metformin	Shigiyama et al. (2017)
Dapagliflozin	II	Metabolic modifiers	Dapagliflozin reduced ahres and improved oxidative stress/mitochondrial function	54	3 months	Dapagliflozin vs. Placebo	Nantsupawat et al. (2024)
Empagliflozin	II	Metabolic modifiers	Reduced myocardial glucose uptake and resting blood flow	13	4 weeks	Empagliflozin vs. placebo	Lauritsen et al. (2021)
Empagliflozin	II	Metabolic modifiers	Empagliflozin improved oxidative stress markers and nyha functional class	80	12 weeks	Empagliflozin vs. Placebo	Eshraghi et al. (2025)
Statins							
Atorvastatin	III	Chronic Secondary Prevention	Atorvastatin failed to prevent heart function decline in patients receiving anthracyclines	202	Up to 24 months	Atorvastatin vs. Placebo	Makhlin et al. (2024)
Atorvastatin, rosuvastatin	II	Chronic Secondary Prevention	Both statins effectively lowered lipids and improved oxidative stress	52	8 weeks	Atorvastatin vs. Rosuvastatin	Tonu et al. (2019)
Icosapent ethyl	II	Chronic Secondary Prevention	Icosapent ethyl reduced oxidative stress	70	3 months	Icosapent ethyl vs. usual care	Bakbak et al. (2024)
Atorvastatin	II	Chronic HF	Atorvastatin reduced oxidative stress marker	16	30 days	Atorvastatin vs. Placebo	Iacovelli et al. (2024)
Simvastatin	II	Chronic Secondary Prevention	Simvastatin significantly decreased oxidative stress marker	56	3 months	Simvastatin vs. no statin	Kruk et al. (2025)
Nitrates							
Dietary nitrate/nitrite	II	Metabolic modifiers	Dietary nitrate/nitrite supplementation increased vo2max and improved skeletal muscle oxidative capacity	36	8 weeks	Beetroot juice with nitrate/nitrite vs. Placebo	Turner et al. (2022)
Dietary nitrate	II	Chronic Secondary Prevention	Results pending	25	Acute dose +1 week daily	Nitrate-rich vs. Nitrate-depleted beetroot juice	Benjamim et al. (2023)
Dietary nitrate	II	Chronic Secondary Prevention	Dietary nitrate intake subtly improved redox balance and reduced classical monocytes	15	4 weeks	Nitrate-rich vs. Nitrate-depleted beetroot juice	Fejes et al. (2024)
Dietary nitrate + vitamin c	II	Chronic Secondary Prevention	Combining dietary nitrate and vitamin c significantly reduced daily systolic bp and increased nitrate/nitrite markers	10	Variable	Nitrate + vitamin c, nitrate alone, nitrate-depleted	Lbban et al. (2024)

Vitamins C and E, acting as water-soluble (C) and lipid-soluble (E) antioxidants respectively to scavenge ROS and protect membranes (Kalogerakou and Antoniadou, 2024; Alberts et al., 2025), have shown mixed clinical results. Large trials of high-dose Vitamin E, such as the HOPE trial, failed to show cardiovascular benefit and even suggested potential harm, possibly due to complex metabolism, potential pro-oxidant effects in chronic settings, or insufficient targeting to acute injury (Lonn et al., 2005). Conversely, smaller studies exploring acute, peri-procedural administration, such as intravenous Vitamin C combined with E pre-PCI [PREVEC trial (Rodrigo et al., 2014)] or intracoronary Vitamin E during surgery (Canbaz et al., 2003), showed some positive signals regarding reduced infarct size or injury markers, underscoring the importance of timing and local delivery. Indeed, the failure of long-term, high-dose Vitamin E in the HOPE trial offers a classic clinical example of disregarding the principles of redox hormesis discussed in Section 2.2. Instead of restoring balance, this non-specific scavenging strategy likely blunted essential, low-level ROS signals required for activating endogenous protective pathways, paradoxically leaving the myocardium more vulnerable.

N-acetylcysteine (NAC), a glutathione precursor that enhances endogenous defense and acts as a direct scavenger (Orhan et al., 2006; Pasupathy et al., 2017), has demonstrated more consistent acute benefits. Phase II trials showed reduced injury markers during surgery (Orhan et al., 2006) and, notably, a significant reduction in infarct size and improved myocardial salvage when combined with nitrates in acute STEMI patients undergoing primary PCI [NACIAM trial (Pasupathy et al., 2017)], suggesting potential utility in the acute setting, possibly by enhancing endogenous defenses. However, its broader clinical utility remains unproven, with inconsistent results across studies likely stemming from fundamental strategic flaws. Firstly, the delivery method: existing trials have universally relied on systemic (oral or IV) administration, a route likely too slow and diffuse to counter the rapid oxidative burst in the reperfusing heart, while a more direct intracoronary approach has never been tested. Secondly, an emerging safety concern: NAC’s long-held safety profile has been challenged by preclinical evidence that it can paradoxically promote tumor angiogenesis (Wang T. et al., 2023). These unaddressed issues of suboptimal delivery and potential off-target toxicity provide a compelling explanation for NAC’s translational failure to date. The case of NAC encapsulates the core challenges of MIRI therapy: while its mechanism of enhancing endogenous defenses is conceptually superior to direct scavenging (as argued in Section 2.3), its clinical potential is ultimately nullified by the formidable practical hurdles of delivery and timing required to counteract the rapid and localized oxidative burst (Section 2.1).

While the mitochondrial antioxidant Coenzyme Q10 (CoQ10) showed promise in the Q-SYMBIO trial (Mortensen et al., 2014), its clinical translation is severely constrained by profound pharmacokinetic hurdles. The combination of poor oral bioavailability and a saturable absorption mechanism makes it exceptionally difficult for standard formulations to achieve and sustain therapeutic plasma concentrations reliably. This fundamental delivery challenge is a key factor behind the inconsistent clinical outcomes reported for CoQ10 and underscores the critical necessity of advanced formulations, such as nano-emulsions or the use of ubiquinol, to unlock its true therapeutic potential.

Edaravone, a synthetic free radical scavenger, showed potential by reducing reperfusion injury markers in a Phase II acute MI clinical trial (Tsujita et al., 2006). However, despite this early promise and supportive preclinical data, edaravone has failed to become a standard therapy for myocardial reperfusion injury. Its clinical adoption is significantly hampered by practical limitations, including an intravenous-only administration route unsuitable for long-term or outpatient care and a narrow therapeutic window. Furthermore, its current regulatory approval is restricted to neurological conditions like ALS, creating substantial off-label barriers. Crucially, its efficacy in large-scale cardiovascular outcome trials remains unproven, leaving it as a theoretically interesting but clinically unvalidated option in an era dominated by well-established, evidence-backed reperfusion strategies.

Conversely, Melatonin, investigated for its potential antioxidant effects, failed to demonstrate significant improvement in outcomes for STEMI patients undergoing PCI in the MARI trial (Dominguez-Rodriguez et al., 2017), highlighting that effectiveness varies among antioxidants and depends on their specific properties and targets.

The overall mixed clinical results for exogenous antioxidants underscore significant challenges in translating preclinical success, largely contributing to their “unmet promise.” These hurdles include poor pharmacokinetics and bioavailability, leading to insufficient concentrations at the injury site, and a critical lack of specificity. Many exogenous antioxidants fail to selectively target the most relevant oxidants or critical cellular compartments (e.g., mitochondria) where ROS are primarily generated. This non-specific action can also interfere with the physiological signaling roles of ROS (as discussed in Section 2.2), potentially leading to unintended adverse effects or a lack of targeted efficacy. Furthermore, inappropriate dosing or timing relative to the dynamic MIRI process, coupled with inherent patient heterogeneity, further complicates their clinical utility (Lonn et al., 2005; Dominguez-Rodriguez et al., 2017). These limitations collectively emphasize the urgent need for more targeted and sophisticated approaches.

Beyond these, a vast number of natural compounds have demonstrated preclinical antioxidant and cardioprotective potential in MIRI models. These include resveratrol (Shahcheraghi et al., 2023), curcumin (Cox et al., 2022), quercetin (Bellavite et al., 2023), and epigallocatechin gallate (Mohd Sabri et al., 2022), which often act through multiple mechanisms including direct scavenging and activation of pathways like Nrf2. However, their translation is hampered by poor bioavailability and a lack of robust clinical trials in the acute MIRI setting.

### 3.2 Enhancing endogenous antioxidant defense

Recognizing the limitations of directly scavenging the massive and dynamic oxidative burst with exogenous antioxidants, enhancing the heart’s intrinsic antioxidant defense mechanisms represents a potentially more effective and sophisticated strategy, directly addressing the functional failure of these systems during MIRI. The Nrf2 pathway is the master regulator of cellular antioxidant and detoxification responses, making it a prime target for bolstering endogenous protection against oxidative stress in MIRI. The Nrf2 pathway operates *via* a Keap1-mediated mechanism where oxidative stress triggers Nrf2 release, allowing its nuclear translocation and binding to antioxidant response elements (Shen et al., 2019). This upregulates a wide array of protective genes, including enzymatic antioxidants (e.g., HO-1, NQO1) and components of GSH synthesis, significantly enhancing cellular capacity to neutralize oxidants. Numerous preclinical studies with pharmacological Nrf2 activators (e.g., sulforaphane, naringenin, curcumin) have demonstrated significant cardioprotection in MIRI models, attenuating injury, reducing infarct size, and decreasing oxidative stress, inflammation, and cell death (Wang R. et al., 2023; Cui et al., 2025). These benefits are often linked to enhanced antioxidant capacity and potential crosstalk with other protective pathways (Liu P. et al., 2021; Xu et al., 2021; Wang et al., 2022). Despite compelling preclinical evidence, clinical translation of Nrf2 activators for MIRI remains in its infancy, with a significant lack of published trial data specifically in this acute setting (summarized in Table 1). While an ongoing Phase II trial is evaluating sulforaphane (NCT05408559), results are not yet available, highlighting the limited clinical evidence.

Key challenges hindering translation include identifying activators with optimal safety and pharmacokinetic profiles that allow for precise, selective, and temporal activation of Nrf2. This is paramount to maximize cardioprotection while minimizing potential off-target effects. Specifically, while Nrf2 activation is protective in acute MIRI, its sustained or non-selective overactivation may promote cancer cell proliferation and drug resistance by regulating the expression of antioxidant genes, thereby helping cancer cells resist oxidative damage caused by chemotherapy drugs and enhancing their survival capabilities (Nezu and Suzuki, 2021; Nguyen et al., 2025). Furthermore, determining the effective dose and therapeutic window, the lack of reliable biomarkers to confirm Nrf2 activation and predict response, and the need for better patient selection are also critical. This translational challenge highlights the inherent risks of manipulating master regulators. Potential off-target effects, such as promoting cancer, serve as a crucial reminder that even protective pathways have context-dependent effects, echoing the delicate balance of redox signaling *versus* distress laid out in Section 2.2.

Beyond Nrf2, the JAK2/STAT3 signaling pathway represents another crucial component of the heart’s endogenous defense against MIRI. As discussed in Section 2.3, this pathway is influenced by oxidative stress and plays a protective role in the acute phase by promoting cardiomyocyte survival and upregulating anti-apoptotic and antioxidant proteins (Liu P. et al., 2021; Xiong et al., 2021). Preclinical studies suggest that activation of the JAK2/STAT3 pathway contributes to the cardioprotective effects of various agents (Mahdiani et al., 2022), and some compounds may exert their benefits partly through this mechanism (Xiong et al., 2021). This positions JAK2/STAT3 modulation as a potential strategy for enhancing endogenous cardioprotection against oxidative damage. Despite this preclinical promise, clinical translation specifically targeting JAK2/STAT3-mediated oxidative stress as the primary therapeutic strategy in acute myocardial ischemia is notably lacking, highlighting the need for more focused clinical investigation. Therefore, realizing the significant theoretical promise of enhancing endogenous antioxidant defense *via* pathways like Nrf2 and JAK2/STAT3 requires extensive further research, particularly well-designed clinical trials incorporating biomarkers to monitor pathway activation and patient response.

### 3.3 Strategies targeting downstream pathological consequences of oxidative stress

Given that oxidative stress initiates and exacerbates a cascade of downstream pathological events, therapeutic strategies can also focus on interrupting these consequences, even if the primary oxidative insult is not fully neutralized.

#### 3.3.1 Anti-inflammatory agents

Given that oxidative stress is a powerful trigger for inflammation, forming a vicious cycle of tissue damage, targeting the inflammatory cascade is a rational, albeit indirect, strategy to mitigate the downstream consequences of the initial oxidative insult. Targeting the inflammatory response represents a crucial therapeutic avenue. Various anti-inflammatory agents have been investigated, broadly categorized into agents with general anti-inflammatory effects and those targeting specific mediators or pathways.

##### 3.3.1.1 Broad anti-inflammatories

Encompass agents that exert relatively non-specific anti-inflammatory actions. Examples include nonsteroidal anti-inflammatory drugs (NSAIDs) and corticosteroids. While these drugs can reduce inflammation, their clinical utility in acute MI is significantly hampered by substantial side effects, such as increased cardiovascular risk (particularly with certain NSAIDs) and systemic immunosuppression (with corticosteroids) (Ju et al., 2023). These agents often lack specificity for the precise inflammatory pathways driving MIRI, leading to a poor risk-benefit profile and inconsistent efficacy in clinical trials for this acute setting. Colchicine, which inhibits microtubule polymerization and inflammasome activation, can also be considered in this category due to its multifaceted anti-inflammatory actions, although its mechanism involves specific targets like the NLRP3 inflammasome, a key mediator of oxidative stress-induced inflammation. Notably, low-dose colchicine has demonstrated significant clinical benefits in reducing cardiovascular events in patients with chronic coronary disease and after acute MI in trials like LoDoCo2 and COLCOT, respectively (Tardif et al., 2019; Nidorf et al., 2020). This suggests that modulating inflammation, even through agents with broader mechanisms like inflammasome inhibition, can be clinically effective. However, despite some success with agents like colchicine, the general class of broad anti-inflammatories often lacks specificity for the precise inflammatory pathways driving MIRI, leading to a poor risk-benefit profile and inconsistent efficacy in clinical trials for this acute setting. Given these limitations, strategies targeting specific inflammatory mediators are considered a more promising therapeutic approach.

##### 3.3.1.2 Targeting inflammatory mediators/pathways

More targeted approaches focus on specific cytokines or signaling pathways activated by oxidative stress and inflammation. Examples include inhibitors of IL-1β, IL-6, or components of the NF-κB pathway. Natural compounds like midazolam, vitamin B12, metformin, Cangrelor, and hesperidin have also shown anti-inflammatory effects in preclinical MIRI models, often by modulating macrophage polarization or inhibiting inflammatory pathways like NF-κB (Huang et al., 2020; Xu et al., 2024). Clinical trials evaluating anti-inflammatory agents in MIRI have yielded mixed but overall more encouraging results compared to broad antioxidant supplementation (summarized in Table 1).

##### 3.3.1.3 Targeting interleukins

Tocilizumab, an IL-6 receptor inhibitor, showed a significantly higher myocardial salvage index but no significant difference in final infarct volume compared to placebo (Broch et al., 2021). This suggests potential benefit in limiting early damage but highlights the complexity of translating salvage to reduced final infarct size.

Anakinra, an IL-1 receptor antagonist, demonstrated a significant reduction in 1-year death or new-onset heart failure in STEMI patients (Abbate et al., 2022). This positive outcome suggests that targeting the IL-1 pathway, a key downstream effector of NLRP3 inflammasome activation (which is triggered by oxidative stress), can improve long-term clinical outcomes.

Canakinumab, an interleukin-1β inhibitor, in patients with prior MI and elevated CRP, provided strong evidence for a significant reduction in the risk of recurrent MI, stroke, and cardiovascular death (Ridker et al., 2017). While not specifically in the acute MIRI setting, this trial strongly supports the concept that targeting inflammation, particularly the IL-1β pathway, is beneficial in secondary prevention of atherosclerotic cardiovascular events, which are linked to chronic oxidative stress and inflammation.

However, not all anti-inflammatory strategies have succeeded. The CIRT trial found that low-dose methotrexate did not reduce the risk of cardiovascular events (Ge et al., 2024), and the SOLID-TIMI 52 trial with darapladib (an Lp-PLA2 inhibitor) showed no reduction in major coronary events after acute coronary syndrome (O’Donoghue et al., 2014).

The varied success of anti-inflammatory agents suggests that targeting specific inflammatory pathways (like IL-1β or IL-6) or using agents with broad but relevant mechanisms (like colchicine affecting inflammasomes and neutrophil function) may be more effective than general immunosuppression (methotrexate) or targeting single enzymes (darapladib). The timing and duration of anti-inflammatory therapy relative to the dynamic inflammatory response in MIRI are also likely critical factors. Furthermore, patient selection based on inflammatory biomarkers (like CRP in CANTOS) might be important for identifying responders. Collectively, the varied success within this class powerfully illustrates that merely being ‘anti-inflammatory’ is insufficient. The positive outcomes of agents like canakinumab and colchicine suggest that effective strategies must precisely target the key nodes—such as the IL-1β pathway or the NLRP3 inflammasome—that directly perpetuate the vicious cycle between oxidative stress and inflammation detailed in Section 2.4.1. The failure of broader or less specific agents, therefore, reinforces the central theme that therapeutic success in MIRI demands a highly targeted approach that addresses the specific downstream consequences of the initial oxidative insult.

#### 3.3.2 Anti-cell death agents

In addition to fueling a damaging inflammatory cycle, unchecked oxidative stress directly triggers multiple cell death pathways. Therefore, preventing cardiomyocyte death is paramount for preserving myocardial function after MIRI. Initially, strategies targeted apoptosis, as oxidative stress is a known trigger of the mitochondrial apoptotic pathway. However, clinical trials specifically designed to test direct anti-apoptotic agents in acute MIRI have been largely disappointing (summarized in Table 1).

A precise understanding of these failures requires acknowledging the complexity of cell death in MIRI. It has been shown that the predominant form of cell death following reperfusion is necrosis, whereas apoptosis accounts for only a small percentage of the overall cell death. This dominance of necrosis may explain why anti-apoptotic therapies have shown limited success; for instance, neither the mitochondria-targeting peptide Bendavia nor erythropoietin demonstrated significant benefits in clinical trials (Minamino et al., 2018; Hortmann et al., 2019). Consequently, regulators of the mitochondrial permeability transition pore (mPTP), considered a key driver of necrosis, became the next major therapeutic target.

Cyclosporine A (CsA) was introduced as an mPTP inhibitor, acting indirectly by inhibiting cyclophilin D (CypD) to prevent pore opening. While early, smaller trials showed promise by reducing infarct size (Piot et al., 2008), the larger CIRCUS trial subsequently failed to demonstrate improved clinical outcomes (Cung et al., 2015). This translational failure is likely multifactorial, stemming from CsA’s indirect mechanism, which may permit pore opening under high stimulation, confounding off-target effects like calcineurin inhibition, and the reality that, much like the challenge facing anti-apoptotic strategies, MIRI activates multiple redundant cell death programs (such as the increasingly recognized ferroptosis), making the inhibition of mPTP-mediated necrosis alone likely insufficient to salvage the myocardium (Zhou et al., 2020). The consistent clinical failure of agents targeting a single mode of cell death provides compelling clinical validation for the mechanistic principle outlined in Section 2.4.2: MIRI triggers an interconnected web of cell death programs, suggesting future strategies may need to be multi-modal or target more upstream events.

### 3.4 Clinically successful multi-targeted agents with pleiotropic antioxidant effects

Beyond strategies primarily focused on directly modulating oxidative stress or its immediate downstream consequences, certain established or emerging cardioprotective agents have demonstrated clinical success in MIRI, at least in part, through mechanisms that involve mitigating oxidative stress alongside other pathways. Their success may offer valuable insights into overcoming the ‘unmet promise’ of single-target antioxidant therapies. These agents often possess multi-targeted actions, addressing several facets of MIRI pathophysiology simultaneously.

#### 3.4.1 SGLT2 inhibitors

Sodium-glucose co-transporter 2 (SGLT2) inhibitors, initially developed for diabetes management, have demonstrated remarkable cardiovascular benefits, including reduced hospitalization for heart failure and cardiovascular death, even in patients without diabetes. While their primary mechanism involves inhibiting glucose reabsorption in the kidney, their cardioprotective effects in MIRI are thought to be mediated by multiple pathways, including a significant contribution from reducing oxidative stress and inflammation (Pham et al., 2024; Armillotta et al., 2025). These antioxidant effects are thought to be mediated through a dual mechanism: indirectly, by improving systemic metabolism and cardiac energy efficiency (Usman et al., 2024), and more directly, through the inhibition of myocardial NOX2 expression and the activation of endogenous antioxidant pathways such as Nrf2(Preda et al., 2024).

Preclinical studies have shown SGLT2 inhibitors can decrease ROS production, enhance antioxidant defense, and suppress inflammatory responses in the myocardium (Harris et al., 2024; Pham et al., 2024; Armillotta et al., 2025). Clinical evidence, as summarized in Table 1, includes studies showing that Empagliflozin improved oxidative stress markers and NYHA functional class in T2DM patients with HFrEF (Eshraghi et al., 2025), and Dapagliflozin reduced the number of AHREs and improved oxidative stress/mitochondrial function in the clinical AF subgroup of patients with CIEDs (Nantsupawat et al., 2024). Dapagliflozin also improved endothelial function and reduced oxidative stress in patients with early-stage T2DM (Shigiyama et al., 2017) and improved endothelial function compared to glibenclamide in T2DM patients with subclinical atherosclerosis (Sposito et al., 2021). Another study explored Empagliflozin’s effects on myocardial glucose uptake and blood flow (Lauritsen et al., 2021). While large outcome trials were not specifically focused on acute MIRI, these studies in Table 1 provide clinical evidence of SGLT2 inhibitors influencing oxidative stress and related parameters in various patient populations.

#### 3.4.2 Statins

HMG-CoA reductase inhibitors (statins) are cornerstone therapies for preventing and treating atherosclerotic cardiovascular disease. While their primary mechanism is cholesterol lowering, statins also possess pleiotropic effects, including anti-inflammatory and antioxidant properties (Iqbal et al., 2019). Statins can reduce oxidative stress by inhibiting NOX activity, enhancing eNOS function, and upregulating antioxidant enzymes. These effects contribute to improved endothelial function, reduced inflammation, and potentially direct cardioprotection in ischemic conditions (Chen W.-H. et al., 2024). Clinical trials, as shown in Table 1, have investigated the effects of statins on oxidative stress markers in various patient populations. For instance, Atorvastatin and Rosuvastatin significantly improved oxidative stress markers and lipid profiles in hyperlipidemic patients (Tonu et al., 2019). Atorvastatin also improved microvascular reactivity and reduced the oxidative stress marker malondialdehyde in patients with HFpEF (Iacovelli et al., 2024), and Simvastatin significantly decreased protein carbonylation (an oxidative stress marker) in COPD patients. While Atorvastatin had modest effects on oxidative/nitrosative stress biomarkers in the PREVENT trial involving patients receiving anthracyclines (Makhlin et al., 2024), these studies collectively support the concept that statins influence redox balance. Additionally, Icosapent ethyl, listed under this category in Table 1, modulated vascular regenerative cells and reduced oxidative stress in progenitor cells in patients with hypertriglyceridemia (Bakbak et al., 2024). These rapid pleiotropic effects, including their influence on redox balance, contribute to their standard use in the acute MI setting (Wu et al., 2024).

#### 3.4.3 Nitrates

Organic nitrates like nitroglycerin are vasodilators widely used in ischemic heart disease to improve myocardial oxygen supply by dilating coronary arteries. Beyond vasodilation, nitrates can also exert antioxidant effects, for instance, by scavenging free radicals or improving eNOS function (Zhu et al., 2021). Clinical studies listed in Table 1, primarily focusing on *dietary* nitrate/nitrite supplementation, have shown effects such as subtly shifting redox balance towards a less pro-oxidative profile and decreasing classical monocytes in hypertensive patients, significantly lowering daily systolic BP and increasing nitrate/nitrite markers when combined with vitamin C in healthy young adults (Lbban et al., 2024), and increasing VO2max and improving skeletal muscle oxidative capacity in T2DM (Turner et al., 2022). A study protocol is also listed investigating dietary nitrate effects on cardiovascular performance, endothelial function, and oxidative stress during and after exercise in postmenopausal women with hypertension (Benjamim et al., 2023). These studies suggest that modulating the nitrate-nitrite-NO pathway, including through dietary intake, can influence redox status, although clinical trials with organic nitrates specifically assessing antioxidant effects in acute MIRI are less represented in Table 1.

**FIGURE 5:**
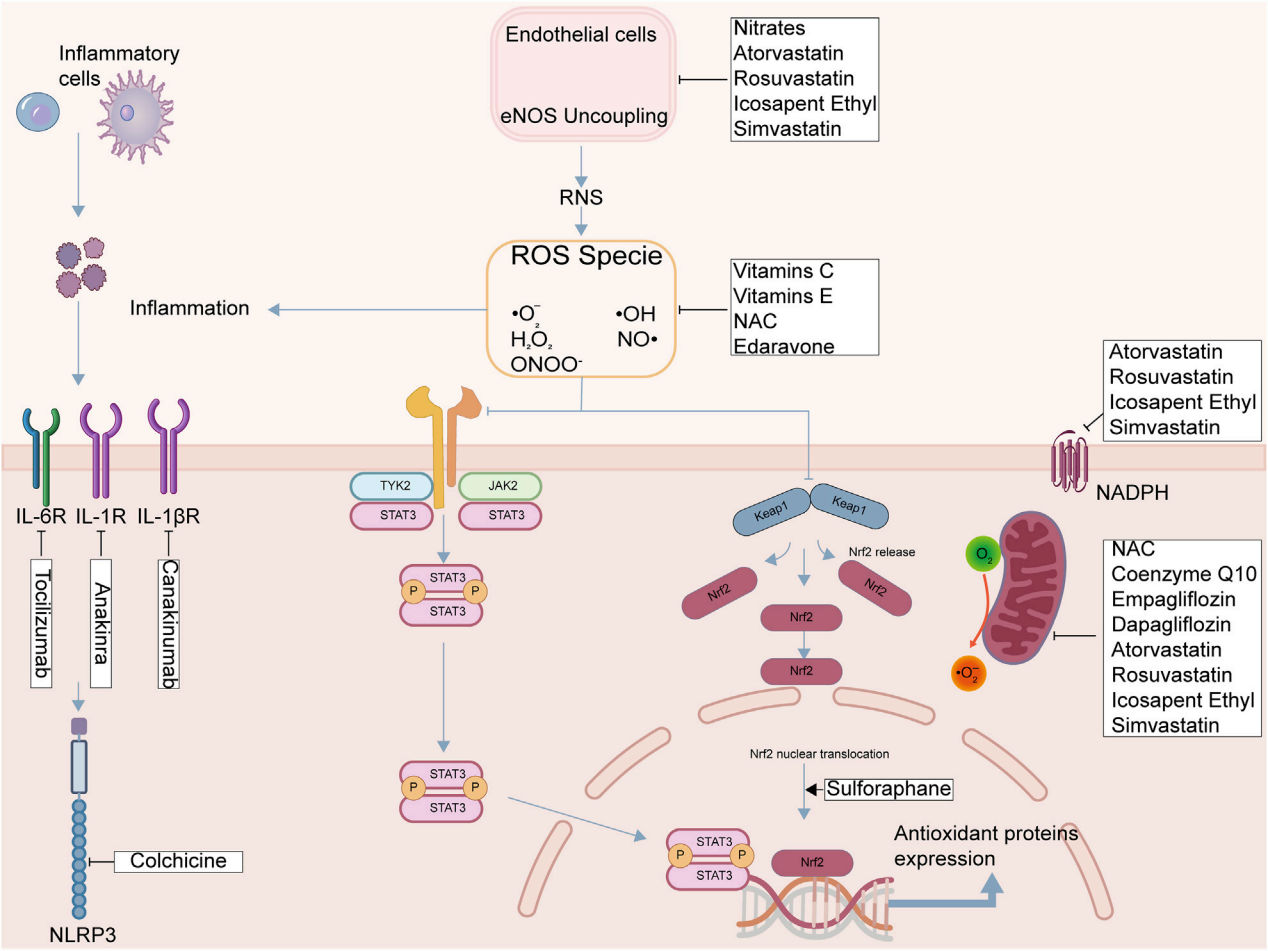
Mechanisms of therapeutic agents targeting oxidative stress in MIRI. Strategies include: (1) direct ROS scavenging (e.g., Vitamins C, E); (2) inhibiting ROS/RNS sources (e.g., Statins, Nitrates); (3) targeting downstream inflammation (e.g., Canakinumab, Colchicine); and (4) enhancing endogenous defenses (e.g., Sulforaphane).

These multi-targeted agents highlight that effective cardioprotection in MIRI may require simultaneously addressing multiple pathological pathways, including oxidative stress, inflammation, and metabolic dysfunction. While their primary indications and mechanisms may vary (Figure 5), their ability to favorably influence the redox balance, as supported by the clinical studies on biomarkers and surrogate endpoints listed in Table 1, contributes to their overall beneficial effects. The clinical evidence for some of these agents (like SGLT2 inhibitors and statins) in improving cardiovascular outcomes is strong, supporting the concept that modulating pathways intertwined with oxidative stress is a viable therapeutic approach. However, further research is needed to fully elucidate the contribution of their antioxidant effects to clinical outcomes in acute MIRI and to explore their optimal use in this specific context. The clinical evidence for the diverse strategies discussed above, ranging from direct antioxidants to multi-targeted agents, is summarized in Table 1, which illustrates the varied outcomes and highlights the ongoing translational challenges.

### 3.5 Overcoming delivery barriers with advanced therapeutic systems

A significant challenge contributing to the ‘unmet promise’ of many promising preclinical antioxidant and other oxidative stress-modulating agents is the inefficient and non-targeted delivery of these compounds to the injured myocardium (Borrelli et al., 2021). Systemic administration often results in low drug concentrations at the site of injury, rapid clearance, and potential off-target effects. Novel drug delivery systems offer powerful solutions to overcome these limitations by enhancing drug bioavailability, improving targeting specificity to the ischemic/reperfused tissue, and controlling drug release kinetics (Shi et al., 2022).

#### 3.5.1 Nanoparticle-based delivery

Nanocarriers, such as liposomes, polymeric nanoparticles, and micelles, are at the forefront of MIRI therapy. They can encapsulate a wide array of therapeutic agents, including natural compounds (e.g., Notoginsenoside R1), small molecule inhibitors, and gene therapies targeting oxidative stress pathways (Li H. et al., 2022; Ji et al., 2023). Nanoparticles protect their cargo from degradation, improve solubility, and favorably alter pharmacokinetics. A key strategy involves designing systems responsive to the MIRI microenvironment, such as nanoparticles that release their payload in response to high levels of ROS (Chang et al., 2024; Zuo et al., 2025).

By engineering their surface properties, nanoparticles can be designed for passive accumulation in the injured myocardium or active targeting of specific cell types. This targeted delivery significantly increases local drug concentration while minimizing systemic toxicity. For instance, engineered neutrophil membrane-mimicking nanocomposites have shown significant efficacy by leveraging integrin-mediated targeting (Jiang et al., 2025). Similarly, biomimetic systems like exosomes and nanozymes enhance therapeutic efficacy through their inherent biocompatibility (Jin et al., 2024; Geng et al., 2025).

#### 3.5.2 Cell-based and exosome delivery

Utilizing cells, such as mesenchymal stem cells (MSCs) embedded within biomaterial patches, as carriers for therapeutic factors is another promising approach (Sharma et al., 2022). These cells can home to the injury site, providing sustained local delivery of antioxidant and regenerative support.

Building on this, exosome-based systems are emerging as a superior solution, capitalizing on their role as natural nanocarriers with low immunogenicity (Yuan et al., 2018; Chi et al., 2025). Exosomes can deliver protective cargo, like ncRNAs (e.g., miR-126), to directly mitigate oxidative stress (Wang et al., 2019). The future of this field lies in engineering these vesicles for enhanced efficacy. Advanced strategies include modifying their surface with cardiac-targeting peptides to improve homing and delivering highly advanced cargo, such as CRISPR-Cas9 ribonucleoproteins for precise cardiac genome editing (Mun et al., 2024).

#### 3.5.3 Targeting ligands and advanced biomaterials

To further enhance precision, attaching targeting ligands (e.g., peptides, antibodies) to nanocarriers has proven highly effective. Examples include using CD11b antibodies to target inflammatory cells or folic acid to bind activated macrophages at the infarct site (Ma et al., 2025).

Injectable hydrogels and advanced biomaterials offer another dimension of control, providing a localized depot for the sustained release of therapeutics. These systems can be designed to be “paintable” for easy application on the epicardial surface or as microneedle patches for sequential, multi-drug delivery aligned with the pathological phases of MIRI (Lee et al., 2024; He et al., 2025). Injectable microporous scaffolds can also facilitate tissue ingrowth while releasing anti-fibrotic and pro-angiogenic drugs (Fang et al., 2020). These advanced systems ensure that therapeutic agents reach the myocardium at sufficient concentrations and for an adequate duration, representing a critical step in translating promising science into effective treatments.

#### 3.5.4 The ‘Bench-to-Bedside’ translational gap

While these advanced delivery systems show immense preclinical promise, their clinical translation faces significant hurdles. The primary challenge lies in manufacturing and quality control. Reproducing complex, multi-component systems—such as hybrid cell membrane-coated nanoparticles or hydrogels with precisely tuned degradation and sequential drug release profiles (He et al., 2025)—at a clinical scale under Good Manufacturing Practice (GMP) standards is a formidable task. Ensuring batch-to-batch consistency in particle size, drug loading efficiency, surface chemistry, and release kinetics is critical for predictable therapeutic outcomes and regulatory approval.

Furthermore, immunogenicity remains a critical safety concern. While endogenous carriers like extracellular vesicles or biomimetic coatings are designed to minimize immune recognition, the introduction of targeting ligands, synthetic polymers, or residual manufacturing components can provoke unintended inflammatory or foreign body responses, potentially neutralizing the therapeutic effect or causing adverse events. Predictive computational tools are emerging to de-risk these platforms, but thorough *in vivo* assessment is indispensable (Cun et al., 2025). Finally, navigating the regulatory landscape for these combination products (drug/biologic + device) is inherently complex, requiring extensive data on long-term stability, biodistribution, and the ultimate fate and toxicity of all system components. Overcoming these practical barriers is the final, critical step toward translating these innovative delivery strategies into meaningful clinical benefit for MIRI.

## 4 Challenges, future directions, and the promise of precision medicine

### 4.1 Key challenges in targeting oxidative stress for MIRI treatment

Despite compelling preclinical evidence highlighting the central role of oxidative stress in MIRI pathophysiology, translating these findings into effective clinical therapies has proven remarkably challenging, largely contributing to the ‘unmet promise’ observed thus far. A primary hurdle lies in the inherent complexity and dynamic nature of oxidative stress itself. It involves a diverse array of ROS species generated from multiple sources with distinct reactivities and temporal patterns throughout ischemia and reperfusion. This complexity makes identifying and specifically targeting the most relevant pathways within this vast network difficult, as targeting a single oxidant or source may be insufficient and could potentially interfere with physiological redox signaling, given ROS’s crucial dual roles as signaling molecules (as elaborated in Section 2.2) (Cheng et al., 2024). Indeed, non-selective or excessive scavenging of ROS can disrupt this delicate redox balance, potentially leading to unintended adverse effects by impairing vital physiological signaling pathways, as exemplified by their essential roles in processes like neuronal antioxidant responses, embryonic development, and reproductive function (Gualtieri et al., 2021; Liu L. et al., 2021).

Further challenges relate to the practical aspects of therapeutic intervention. Achieving sufficient and sustained drug concentrations specifically at the site of myocardial injury remains a major obstacle for systemically administered agents due to poor pharmacokinetics, rapid clearance, and lack of specific targeting to affected cells (Duan X. et al., 2023). Moreover, the dynamic nature of oxidative stress dictates a potentially narrow and stage-dependent therapeutic window, making the determination of optimal timing for intervention crucial yet challenging in the clinical setting (Fasnacht et al., 2022).

Significant patient heterogeneity in baseline oxidative stress levels, comorbidities, and genetic predispositions further complicates a one-size-fits-all approach, influencing individual responses to targeted therapies (Zou et al., 2024). A critical bottleneck remains the lack of robust biomarkers to assess myocardial redox status, predict therapeutic response, or stratify patients (Vukašinović et al., 2023). These factors, combined with the complexity of designing clinical trials that adequately account for heterogeneity, timing, and appropriate endpoints, contribute to the difficulty in demonstrating clinical efficacy. Furthermore, oxidative stress is intertwined with other pathologies like inflammation and metabolic disturbances, suggesting that targeting it in isolation may be insufficient (Hernández-Ruiz et al., 2025). Addressing these fundamental challenges is essential for successful clinical translation.

### 4.2 Oxidative stress in different MI subtypes and special populations

The role and impact of oxidative stress in MI vary significantly depending on the specific clinical presentation of MI and the presence of underlying comorbidities, underscoring the fundamental need for tailored therapeutic strategies and a precision medicine approach. In acute MI, particularly reperfused STEMI, the dominant feature is a massive oxidative burst upon reperfusion, superimposed on ischemic stress (Yazdi et al., 2023). While core ROS generation mechanisms are similar in STEMI and NSTEMI, the severity, duration of ischemia, and nature of reperfusion influence the magnitude and kinetics of this burst. In contrast, chronic ischemic heart disease involves prolonged, low-grade oxidative stress contributing to maladaptive remodeling and fibrosis (Gallo et al., 2024; Loffredo and Carnevale, 2024), with ROS sources potentially more linked to chronic inflammation and metabolic alterations, suggesting different therapeutic targets than acute scavenging.

Furthermore, the presence of comorbidities profoundly alters the baseline redox state and inflammatory milieu, influencing MIRI severity and therapeutic response. Diabetic patients exhibit elevated chronic oxidative stress and inflammation (Li et al., 2025), exacerbating MIRI injury with larger infarcts and poorer outcomes (Sztolsztener et al., 2022), potentially due to hyperactive ROS sources and impaired defenses. Chronic hypertension is also associated with increased vascular and myocardial oxidative stress (Loffredo and Carnevale, 2024), priming the heart for more severe ischemic injury. Similarly, patients with pre-existing heart failure (Aimo et al., 2020; Gallo et al., 2024) or those who are aging (Zhang J. et al., 2021) often have elevated chronic oxidative stress and impaired defenses, increasing their vulnerability to acute ischemic insults and reducing recovery capacity.

Understanding these distinct oxidative stress profiles and responses across different MI subtypes and patient populations is critical for moving beyond a generalized approach. The variability introduced by factors like the type of ischemia, diabetes, hypertension, heart failure, and aging necessitates patient stratification and the development of personalized therapeutic strategies. Future approaches should integrate individual risk factors, comorbidities, and potentially biomarkers of oxidative stress to guide the selection and timing of interventions, ultimately aiming to improve outcomes based on each patient’s unique oxidative stress landscape (Vornic et al., 2024).

### 4.3 Future research directions

**FIGURE 6:**
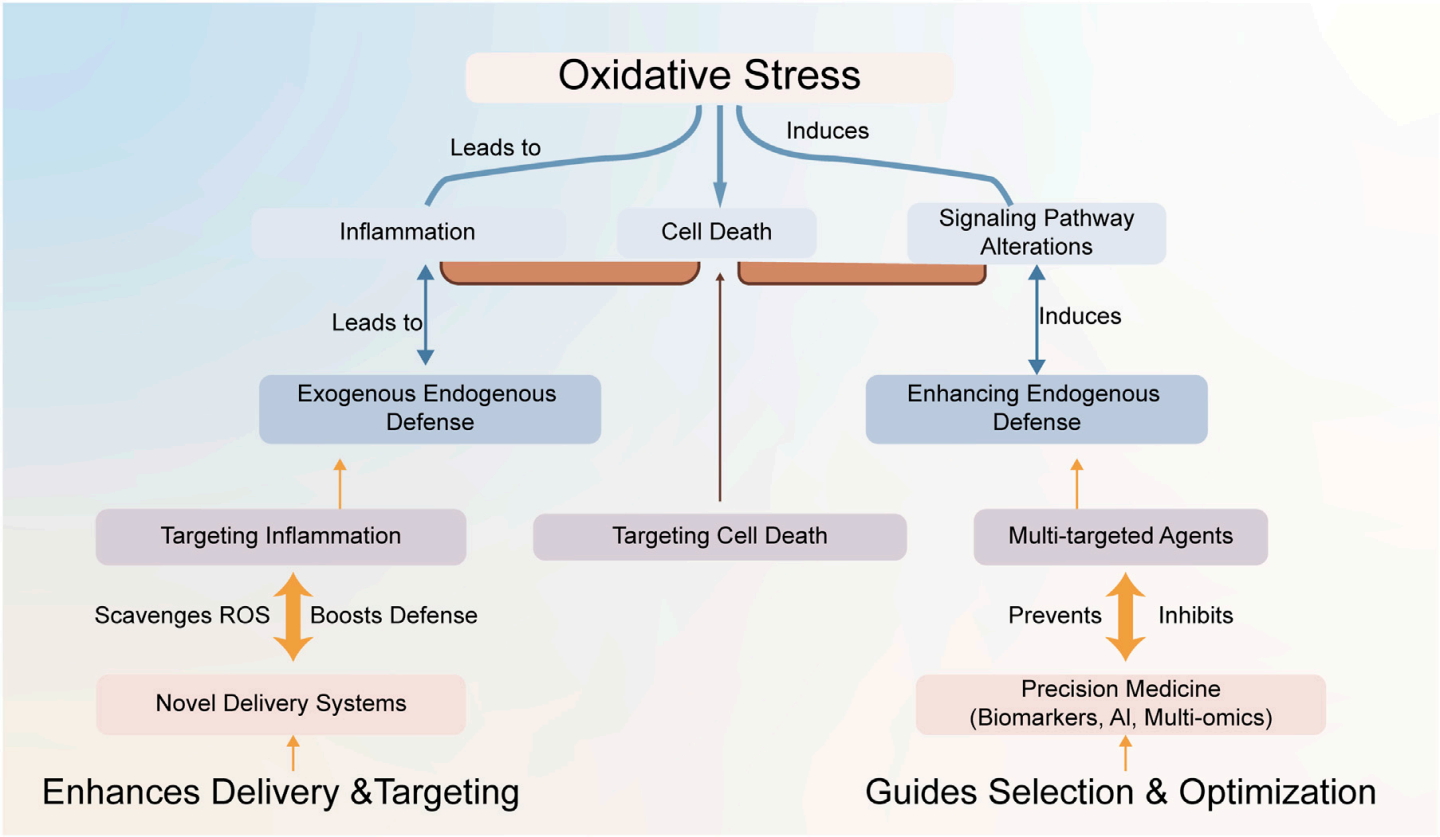
A precision medicine framework for targeting oxidative stress in MIRI. This framework integrates therapeutic strategies targeting inflammation and cell death with multi-targeted agents. The choice of strategy is guided by precision medicine tools (biomarkers, multi-omics, AI) to address the specific pathological drivers of oxidative stress.

Addressing the challenges in translating oxidative stress research into effective MIRI therapies requires concerted future efforts focused on refining mechanistic understanding and developing innovative, targeted interventions. Moving beyond broad antioxidant approaches, research must prioritize more precise target identification and validation within the complex oxidative stress network. This includes investigating the specific roles of different NOX isoforms in distinct cell types, developing strategies to regulate mitochondrial ROS production without compromising function (Romo-González et al., 2023), identifying critical nodes in oxidative stress-sensitive signaling pathways, and exploring the potential of modulating miRNAs involved in redox regulation to achieve highly specific redox modulation (Carbonell and Gomes, 2020). A modular overview of these strategies is presented in Figure 6.

A critical need is the development and validation of robust, dynamic, and specific biomarkers for oxidative stress in MIRI. These biomarkers should reflect real-time redox status, predict therapeutic response, and enable patient stratification. Identifying biomarkers that reflect the activity of specific ROS-generating enzymes or pathway activation is also crucial. Such biomarkers must be validated in large clinical cohorts to determine their utility for early diagnosis, risk stratification, predicting therapeutic response, and monitoring efficacy (Rosenblatt et al., 2025). Furthermore, advancing and clinically translating novel drug delivery systems with enhanced targeting capabilities to the injured myocardium or specific cell types is essential to achieve sufficient local drug concentrations for precise redox modulation.

Given the complex and interconnected nature of oxidative stress and its downstream effects, exploring multi-target combination therapy regimens may be more effective than single-target approaches, aiming for synergistic effects on the ‘oxidative storm’ and its consequences. Research should investigate rational combinations of agents targeting different aspects of oxidative stress or combining them with established MIRI treatments. Integrating multi-omics data and utilizing artificial intelligence can provide deeper mechanistic insights and facilitate personalized approaches by identifying individual variations and optimizing treatment strategies. Ultimately, designing more targeted and effective clinical trials based on improved understanding, utilizing novel delivery systems, incorporating validated biomarkers, and focusing on specific patient subgroups will be critical for successfully translating research findings into improved outcomes for MIRI patients.

### 4.4 The promise of precision medicine based on oxidative stress

The inherent heterogeneity of MIRI patients underscores the critical need for a paradigm shift from a ‘one-size-fits-all’ approach to precision medicine, a path now being paved by tangible technological advancements. At the forefront of this revolution is the integration of artificial intelligence (AI) and machine learning (ML), which are transforming the rapid and accurate stratification of patients. Landmark studies demonstrate that AI-enhanced electrocardiogram analysis can identify high-risk occlusion myocardial infarction even without classic ST-segment elevation, outperforming both clinicians and existing commercial systems (Al-Zaiti et al., 2023; Lee et al., 2025). This diagnostic power extends to biomarkers. For instance, algorithms like CoDE-ACS and ARTEMIS personalize troponin interpretation by integrating clinical features. This approach has more than doubled the efficiency of safely ruling out MI in the emergency department compared to standard guidelines (Doudesis et al., 2023; Toprak et al., 2024). Furthermore, AI-driven analysis of cardiac imaging, from quantifying patient-specific coronary plaque risk on CT angiography to using “ultrasomics” to characterize myocardial texture on echocardiograms, is providing deeper, more predictive insights than ever before, establishing a robust foundation for personalized risk assessment (Miller et al., 2024; Jamthikar et al., 2025).

Accurate stratification must be coupled with a deep understanding of an individual’s unique biological landscape, a feat now achievable through multi-omics and novel biomarkers. This approach moves beyond simply diagnosing an MI to uncovering its specific pathophysiological drivers. For example, untargeted plasma metabolomics can reveal distinct metabolic signatures, such as elevated acylcarnitines, that point directly to the mitochondrial dysfunction and impaired fatty acid oxidation that fuel the oxidative burst in MIRI (Lv et al., 2024). Clinically accessible markers like the stress-hyperglycemia ratio (SHR) further reflect this interplay between systemic metabolic disturbance and oxidative stress, offering powerful prognostic value (Lucci et al., 2025; Song et al., 2025). Critically, integrating genomic and molecular data has uncovered fundamental mechanisms of patient heterogeneity, such as the discovery that a diurnal BMAL1-HIF2A complex regulates myocardial vulnerability to injury, providing a molecular rationale for the long-observed circadian patterns of MI severity and opening the door to “chronopharmacology”—timing interventions for maximum efficacy (Ruan et al., 2025).

Ultimately, personalized diagnosis and phenotyping are only effective if they guide targeted interventions that overcome the historical barriers of drug delivery and non-specificity. Here, precision medicine offers powerful solutions. The emergence of “theranostic” agents, such as the near-infrared dye IR-780, exemplifies this progress; this molecule not only selectively accumulates in ischemic cardiomyocytes for precise imaging but also exerts a direct protective effect by inducing a mitochondrial “quiescent state” that mitigates calcium overload and ROS production (Chen Z. et al., 2024). Concurrently, advanced delivery systems are being engineered to conquer the delivery hurdle. Technologies like ultrasound-targeted microbubble destruction (UTMD) enable the cardiac-specific delivery of gene therapies, enhancing the efficacy of safe, clinically relevant drug doses that would otherwise be insufficient (Wang S. et al., 2023). This synergistic integration—using AI for rapid stratification, multi-omics for deep phenotyping, and targeted systems for precise treatment—forms the definitive framework for translating our understanding of oxidative stress into meaningful clinical benefit and finally fulfilling the ‘unmet promise’ of MIRI therapy.

## 5 Conclusion

MIRI remains a critical clinical challenge, largely driven by oxidative stress. This review has highlighted that in MIRI, excessive ROS production overwhelms the heart’s endogenous defenses, leading to unchecked oxidative damage and severe downstream consequences. While therapeutic strategies targeting oxidative stress are crucial, their clinical translation has largely represented an ‘unmet promise’ due to the complexity of redox biology, delivery challenges, and patient heterogeneity. To overcome these limitations, we advocate for a paradigm shift towards precision medicine. This involves moving beyond simplistic exogenous scavenging to focus on enhancing intrinsic defense systems (e.g., Nrf2, JAK2/STAT3), leveraging novel drug delivery systems for targeted intervention, and exploring multi-target therapies. Ultimately, integrating robust biomarkers, multi-omics, and AI is the most promising path to tailor therapies to individual redox profiles, finally fulfilling the promise of antioxidant strategies and improving MIRI outcomes.
